# Beyond the Queue: Exploring Waiting Practices in the Stories of Patients With Breast Cancer

**DOI:** 10.1111/hex.70086

**Published:** 2024-11-17

**Authors:** Nada Akrouh, Rik Wehrens, Erna Scholtes, Hester van de Bovenkamp

**Affiliations:** ^1^ Department of Health Care Governance, Erasmus School of Health Policy & Management Erasmus University Rotterdam Rotterdam The Netherlands

**Keywords:** patients' experiences, patients' stories, rhythms, thematic analysis, waiting, waiting work

## Abstract

**Background:**

Waiting is an important topic in healthcare debates, mostly discussed in the form of waiting lists and waiting times. In this discourse, the experiential element of waiting stays hidden. Understanding the waiting experiences of patients can help to better understand healthcare waiting practices, which have a large impact on patients.

**Methods:**

We performed a thematic analysis on 12 patients' books of women with breast cancer. We focused on the theme of waiting within these stories, through an abductive analysis.

**Results:**

We identified three themes within the waiting practices of patients with breast cancer: (1) Thickening of time, (2) contaminated time and (3) navigating time. The theme thickening of time highlights waiting moments where time is experienced as moving at a very slow pace with intense emotional impact. The theme of contaminated time highlights the waiting processes as an ongoing component of experiencing illness. The theme of navigating time highlights patients' temporal agency, showing their waiting work in the form of strategies for dealing with practical and emotional aspects of waiting.

**Discussion:**

The waiting experiences of patients provide insights into the burden of waiting, which is partly connected to the way healthcare services are organised and the experience of illness. Understanding these multifaceted experiences of patients helps pinpoint areas for healthcare quality improvement.

**Patient or Public Contribution:**

The choice for the theme and approach of this research, waiting, was developed with a citizen science initiative of collecting patient stories.

## 1 Introduction

Waiting takes a prominent place in healthcare debates. The dominant discourse in these debates can be understood from a logistical point of view. Here, waiting takes a tangible form, measured by waiting lists and times, directing attention towards capacity problems and shortcomings in health service planning [1]. Concurrently, waiting is acknowledged as a pertinent policy concern, prompting an exploration of system solutions to address its implications for the functioning of healthcare systems [2]. Exemplary are the rising tensions in the UK on the long waiting lists and times in the National Health Service (NHS) [3].

This prevailing discourse predominantly relies on quantitative measures as benchmarks for the healthcare system's capacity for understanding waiting. However, our study seeks to take a qualitative approach to waiting by delving deeper into the multi‐faceted nature thereof from the perspectives of patients. Waiting, as our research contends, transcends the enumeration of waiting lists and times, extending into a rich experiential terrain that profoundly influences individuals' lives within and beyond care. For example, the universal act of waiting is intricately intertwined with emotions such as hope, uncertainty and anxiety [4]. Moreover, ethnographic research on waiting within the healthcare context, exemplified by Day's [5] study in oncology departments, sheds light on the functioning of the healthcare system. This research reveals bureaucratic inefficiencies within the NHS by explicating the processes that form barriers to timely care. Ethnographic research in other contexts shows that long waiting times in waiting rooms exacerbate anxiety and that patients appreciate comfortable physical environments while waiting [6, 7]. Mulcahy et al. [8] describe how waiting among Canadian cancer patients highlights the power imbalance between patients and healthcare providers.

Research illuminating experiences of waiting provides valuable insights into the multifaceted nature and intrinsic complexities attached to it [5, 9, 10, 11, 12, 13, 14]. Our study contributes to this body of knowledge by placing emphasis on patients' perspectives and understanding their waiting experiences within their day‐to‐day lives by studying stories of patients with breast cancer. This leads us to the following research question: *How do patients’'temporal experiences of waiting unfold, and which waiting practices do they engage in?*

The narratives of patients, as illuminated by White [15], offer a valuable avenue for exploring temporal experiences and arrangements. Studying these narratives allows us to delve into the intricacies of their waiting experiences and the subsequent consequences. We do this by focusing on the stories of patients with breast cancer in the Netherlands. In the next section, we provide an overview of the concepts of subjective time dimensions, temporal agency, and rhythms to help this endeavour. Consequently, we will describe the methods used in our study. Our analysis shows three main aspects of waiting experiences, which we label ‘thickening of time’, ‘contaminated time’ and ‘navigating time’. In the discussion, we connect these themes, situating them in broader discourses about waiting and care.

### 1.1 Subjective Time Dimensions, Temporal Agency and Rhythms

Time is a critical aspect to consider when exploring the concept of waiting. Waiting, as a specific temporal experience, is characterised by elongation [16]. It occurs within a certain time interval, highlighting an inherently future‐oriented element in the waiting process. Various perspectives exist on the conceptualisation of time, encompassing distinctions such as clock time versus lived time, objective time versus subjective time, and between linear and cyclical time [17]. These categorisations highlight how we can understand time in quite different ways, prioritising quantitative or qualitative dimensions and approaching time as something structured, standardised, and measurable or, alternatively, as something that is experienced as more nuanced, complex, and layered. In this study, we will dive into the qualitative dimensions by focusing on the lived experiences of waiting. According to Blue [18], time is practice, meaning that time is a central constitutive feature of practices. Waiting, in this sense, points us to the importance of waiting practices.

To understand these practices, it is important to understand how patients work with time to better understand their waiting experiences. Individuals are not merely passively confronted with time as it passes but try to actively shape their experience of time [19, 20, 21]. Individuals, including patients, exercise temporal agency, actively engaging in what can be termed as “time work”—the practices undertaken to navigate their temporal experiences [22]. An example of time work is the practice of patients adhering to certain medication treatments, which includes, for instance, strategies created to recognise dose time and keep track of medication intake [21]. Drawing from Flaherty's [23] concept of ‘time work’, we introduce the term ‘waiting work’ to describe the behaviours and attitudes of patients as they navigate periods of waiting.

However, patients are limited in their temporal agency. One's temporal agency is often dependent on the temporal agencies of other people or institutions [22]. Institutional rhythms, as we see in healthcare services, play a pivotal role in shaping these dynamics. Well‐established organisations, exemplified by their institutional rhythms, such as opening hours, working days and how these days are structured in time, exert entraining effects on individuals and their practices [18]. Understanding the influences of these institutional rhythms is essential for comprehending how individuals, including patients, navigate and experience the passage of time within the context of waiting in healthcare. The observation of different rhythms in practice aligns with Lefebvre's concept of polyrhythmia, where at times, rhythms synchronise harmoniously, but at times also appear to clash and disintegrate [18]. Waiting, in this sense, finds itself in a complex interplay of rhythms. When addressing the waiting problems in healthcare systems, understanding where these rhythms clash is important for learning how the burden of waiting can be alleviated.

## 2 Methods

We conducted a qualitative thematic analysis of books written by patients with breast cancer.

### 2.1 Study Context

Patients' stories were selected from patientervaringsverhalen. nl (‘patient stories’). This is a large collection of books written by patients and their loved ones on their experiences. The collection of patient stories, which presently surpasses 6200 books and continues to expand, originates from a citizen science initiative led by Coleta Platenkamp, a patient and sociologist. Motivated by her personal experiences with illness, Platenkamp established a foundation committed to the collection of accounts from patients and their kin [24]. Since 2004, volunteers have gathered diverse forms of these narratives, encompassing books, documentaries and blogs, with the overarching goal of disseminating experiential insights of patients and their families [25].

### 2.2 Data Sampling

In February 2023 we selected the condition ‘breast cancer’, perspective ‘patient’, content type ‘book’ and year ‘2020–2023’ in the search engine. This yielded 32 books. We chose to exclude books that did not extensively narrate the author's personal experience of illness. This meant that books primarily categorised as self‐help or poetry were not considered, as they lacked detailed patient narratives. Also, only works authored by individuals based in the Netherlands were included. Ultimately, we included 12 books that described the experience of patients with care (see Supporting Information: Table S1). The age of the authors at the time of writing ranged from 21 to 67 years, with most women between 40 and 50 years. These books, like diaries, prove to be ‘temporally sensitive’ material [12], containing stories from patients' everyday lives, encompassing their experiences, emotions, and reflections within specific temporal contexts.

### 2.3 Data Analysis

During the coding process, we adhered to the three coding steps outlined by Strauss and Corbin [26]: open coding, axial coding, and selective coding. Atlas.ti 22 facilitated the data analysis. In open coding, we closely examined the text of the books, paying particular attention to moments when patients experienced various forms of waiting. We also focused on the emotions associated with these waiting periods, including anxiety, relief, and hope. Additionally, we were attuned towards words related to waiting and time concepts, such as 'minutes' and 'hours'. In our coding, we specified the contexts for the patients' waiting, such as waiting for treatment or diagnosis, and distinguished between waiting at home versus within care facilities. Furthermore, we incorporated the emotional aspects tied to specific moments, processes, and actions taken by patients. For instance, in certain moments, patients were awaiting treatments or scan results (code example: sitting in waiting rooms: unease), while during the broader processes, they were in a state of anticipation for cancer in their life (code example: waiting for cancer: anxiety about the progress of the disease). Constant comparison, involving the examination of how data could be described using existing codes, facilitated differentiation between books and improved systematic coding. In the final selective coding step, we adopted an abductive approach [27]. This outlines a position in qualitative research that allows for iteratively exploring emerging empirical insights through various theoretical lenses; we utilised this approach to connect insights in relation to waiting experiences to various strands of theoretical literature on the subjective dimensions of time, the notion of ‘time work’ and rhythms. This allowed us to better thematize and make sense of the empirical findings.

## 3 Results

### 3.1 Different Dimensions of Waiting

We identified different temporal dimensions of waiting experiences: thickening time, contaminated time and navigating time. In the subsequent sections, we delve into these three dimensions of waiting in greater detail.

### 3.2 Thickening of Time

In the patients' narratives, waiting moments are depicted with remarkable detail. Despite their brevity in terms of clock time, these moments can hold tremendous significance, capable of altering the perception of time itself and intensifying emotions like fear. It is within these brief interludes that time appears to ‘thicken’; moving at a very slow pace. When looking into these waiting experiences, we observe a clash between experienced time and the rhythm of the hospital. Below, we highlight some of these moments.

One regularly occurring situation in which the ‘thickening’ of time becomes visible is in the moments that patients are waiting for scan results, a frequent occurrence after a cancer diagnosis. The anticipation unfolds at home, transforming the place into a nerve‐wracking waiting room. One of the authors describes one of these moments where she is waiting for the result of a CT scan which was done because she experienced heavy abdominal pain:

Waiting for a result, having to wait a while is devastating. Not in the hospital waiting room, but in your own living room. All day long, from 8 AM to 5 PM Startled by every sound of the phone. Not knowing where you stand is stressful. Not knowing when the results are coming is mental torture. Is there a delay due to Covid? Are there not enough radiologists? *(Trepels, 2022, 60)*

This quote captures a moment 3 days after the scan, during which the patient, accustomed to receiving updates from her physician within 2 days, begins to worry and analyse the situation. The notion of ‘mental torture’ poignantly shows the emotional impact of waiting and how the waiting period is disrupting her life.

In the following account, the patient recounts a distressing experience prompted by unexplained and intense itching on her head. Seeking solace and answers, time unfolds with attempts to reach out to her general practitioner (GP) during a vacation period:

I call my general practitioner because I have an itch on my head. Unexplainable, severe itching, already for days. (…) After I discuss it with my physical therapist, she scares me by mentioning that it could be a side effect of liver cancer. Crisis, so then I call the GP anyway. But well, vacation time, my GP is not there. So I continue calling, [this time] with the oncology clinic. My oncologist is also on vacation. That figures: I never call but when I do, I get stuck with the secretary. After being promised I will be called back I enter a restless weekend. Will there be a metastasis after all? On Monday, no surprise, I am not called back. The frustration is mounting again. I just don't understand how something that is so big for me can disappear in the hospital on a pile with 100 other notes. *(Zoelman, 2022, 53)*

This distressing account, in which the fear of metastases is very much present, shows the clash between experienced time and the rhythm of the hospital, with the patient going through a troublous weekend compounded with stress about the lack of good communication with the hospital. The expressed frustration shows the emotional toll of feeling unheard.

These two waiting moments took place at home, changing the house into a waiting room. Now we move to two examples of waiting in a healthcare setting. The following excerpt shows how a patient describes a waiting moment during a radiological examination:

*‘*The assistant left me alone in the room. It's cold, and the radiologist who was supposed to perform the procedure is taking a long time, which is very boring for me. I sit up on the examination table and athletically jump off to do some yoga exercises to warm up my body. While I'm doing this, a technician enters the room. “Madam, what are you doing? Why aren't you lying on the bed?” she asks… “You need to lie down and wait quietly.” (…) She leaves. I feel the time passing, minute after minute goes by, and there's no sign of the radiologist. Every minute feels like too much. Why should I lie here with my upper body exposed, waiting for the doctor for so long? *(Porru, 2022, 38)*

This extract describes how the patient is expected to adapt in both literal and figurative sense to the rhythm of the clinic. This rhythm appears to conflict with the experience of the patient in this case. On the one hand, the radiologist and the technician are busy with analysing the scan under the presumption that the patient should wait for the procedure to be done. On the other hand, the patient is experiencing these moments as distressing not only because of the time she has to wait but also because she is restricted in how the waiting should be done. The patient's temporal agency is limited in this way, as she cannot engage in waiting work, like doing yoga exercises, during the waiting period.

Patients in the stories sit and wait on multiple occasions, such as in waiting rooms. In the following quote, the patient arrives at the hospital for her appointment which will show the results of her scans to rule out any metastases. The patient and her family left the house 1 h later than planned, as the patients' mother called the hospital to enquire about any delays, and it appeared that the physician had a delay of 1 h:

When we get to the ward, it turns out that in the meantime the doctor is running late for an hour and a half. What a long wait in the waiting room. We don't know what to say to each other either. The only thing that goes through my head is: I Will Die. After more than half an hour, the breast cancer nurse comes to pick us up. We can take our seats, the doctor will be here soon. I try to interpret her facial expressions, but I don't succeed. She looks friendly, but not happy. (…) It feels to me it lasts for another hour, but then the doctor finally arrives. His face too, does not give away anything. *(Visser, 2021, 48)*

Despite the proactive attitude to anticipate any delays, waiting appeared to be inevitable. This quote also shows the emotional intensity of waiting in both the waiting room and the consultation room, with the fear intensifying as time passes. With the patient being desperate to know the results, she tries to read the room by carefully looking at the faces of her nurse and physician.

### 3.3 Contaminated Time

Where waiting moments are actively experienced in objectively short timeframes in cases of thickening of time, ranging from minutes to a few days, the stories also teach us about long‐term waiting processes. These show us that waiting is an ongoing and continuous process that develops in each stage of illness, from the discovery of the cancer to the moment one is out of treatment. One of the authors shows some descriptions of this process by stating the following:

Time has become a contaminated concept. I know that I will be better in time, but I also know that this variant of cancer may return over time. *(Oosterbos, 2023, 21)*

The term ‘contaminated’ suggests that the previously straightforward concept of time is now marred by the uncertainty and anxiety of waiting, creating a complex emotional state as one balances the anticipation of recovery with the looming possibility of a cancer recurrence. The notion of ‘contaminated time’ provides us with an understanding of the varied experiences of patients following a breast cancer diagnosis. This notion, therefore, serves as a useful heuristic to analyse the emotional uncertainties and tensions emerging in the ongoing experience of waiting.

Receiving a complete diagnosis is part of a process that usually takes some time. In the case of Porru, it started with finding a lump, for which she called the GP, who referred her to have a mammogram at the hospital. The moment between finding a lump and seeing the GP is described as follows:

My heart fills with fear in these few minutes and there exist no words or pills that can alleviate this fear. Waiting is unfortunately the only thing left for me to do. *(Porru, 2022, 14)*

Waiting as a process is also seen in the stories as they show how different forms of waiting follow each other:

As long as I needed to wait for the consultation, as quickly it passed. Without wanting to say anything about her findings of the physical examination, the GP refers me to a local hospital. It takes four days before I can make an appointment at the mammapoli. The waiting begins again. *(Porru, 2022, 15)*

The excerpt provided from Porru's work exemplifies the continuous and evolving experience of waiting. Initially, there is the wait for a medical consultation at the GP, which, once concluded, promptly transitions to waiting for an appointment at a mammapoli. This transition from one form of waiting to another highlights how waiting is not an isolated event; it flows from one phase to the next. Furthermore, the quote underscores the enduring nature of waiting. Even when one phase of waiting seemingly concludes, a new phase of waiting begins. After having received the mammogram, Porru was diagnosed with breast cancer. To know more about the tumour, a biopsy and MRI scan were needed, so she had to undergo other research on her body, which required more waiting.

In the patients' stories, waiting shows to be a continuous process, as the women continuously anticipate the cancer in distinct phases of their illness trajectories. The quote below shows how waiting becomes a sort of routine, where waiting at times retreats to the background but sometimes also resurfaces and becomes prominent:

The trip [holiday] is great, but at the same time the realisation is growing that I am not doing much more than taking care of my children and waiting for the next scan. I'm waiting for the cancer. *(Scheffers, 2022, 109)*

This quote shows that Scheffers feels that she is constantly waiting for the cancer because her physician told her that her cancer may come back. Others also describe how they are waiting for the cancer to *leave* their body as they are undergoing treatments. This process is laden with ambiguity, as the hope for a positive outcome clashes with the persistent awareness that treatments may not be as effective as desired. The fear for the latter strengthens when treatment has to be awaited:

In any case, I hope we can start Act 3 [breasts radiation treatments] soon, because I'm terrified that there are microcancer cells slumbering, which are going to cause metastases, with all the doom‐and‐gloom scenarios that entails. I ponder a lot about dying, the future and the damage to my body. *(Oosterbos, 2023, 61)*

Waiting is also about waiting for what comes after treatment. For example, waiting to be able to return to ‘normal life’ when she was out of treatments:

(…) I'm waiting for the visas to be allowed back into the Kingdom of the Well and it's taking a long time. Too long. *(Oosterbos, 2023, 113)*

In her healing process, Oosterbos uses Sontag's [28] metaphor describing the desire to return to 'The Kingdom of the Well,' implying that she felt isolated from society as a patient. This relates to the healing process and her complex reintegration trajectory back to work.

Waiting is different when the cancer cannot be cured. When receiving palliative care, the waiting experience is about living with the haunting presence of the cancer:

Except from my short haircut, my illness is not visible. I am beginning to glow more and more. I am living so much that I hardly have time to sleep. I have so much catching up to do from the last six months. At the same time, I have no idea how long I have left. That's why I'm in a hurry. *(Oskam, 2021, 56)*

This passage vividly portrays the experience of time, particularly the sense of urgency and the pursuit to make up for lost moments, all while being haunted by the uncertainty of what lies ahead.

This longing for a normal life is also filled with its own challenges when one receives palliative care:

I find myself in the eye of the needle. In it, because I'm not crawling through it. It feels like silence before the storm (…) In this relatively stable condition, it is still quite an ordeal to pick up the thread. Around me the world goes on, the attention for the disease fades and people prefer to hear that you are well again. That's good and bad. Good because I have the energy again to participate a little, but bad because it is terribly disappointing, and I keep waiting for the moment when everything becomes normal again. Of course, that moment will not come and although I know it cognitively, my feelings keep asking for it. *(Zoelman, 2022, 27)*

This account shows the ambiguity in feelings and emotions. There is the emotional longing to get back to ‘normal’ when finding oneself in a ‘stable’ state, which is powered by societal expectations of getting better. At the same time, one realises that treatment is aimed at extending life rather than achieving complete remission of cancer.

### 3.4 Navigating Time

Having explored waiting moments, waiting processes and their impacts, we now delve into the waiting work patients engage in, showing their temporal agency. This encompasses not only the practical aspects of navigating time but also dealing with the emotional responses it triggers, such as fear and anxiety. The stories used for our analysis illuminate diverse approaches through which patients empower themselves to oversee and work with the waiting period, which includes the establishment of routines and actively seeking information and knowledge. The role played by healthcare professionals in alleviating the burden of waiting for patients through care and support is highlighted here. Additionally, the narratives reveal how patients, through their cumulative waiting experiences, develop a certain level of expertise in navigating their temporal experiences.

One way of dealing with waiting is attempting to control time. This is practised in different ways, ranging from more active ways of gathering information to embracing ‘surrender’ to the rhythms of the healthcare context. Each of these forms of waiting work is presented below.

After receiving the diagnosis, some patients want to seek information to know what awaits them and have control over the situation. In the following example, we see some actions taken to do this:

Besides consciously enjoying everything I could still do and enjoy at that time, and capturing memorable moments from my life, I immersed myself in the world of breast cancer. I started looking for reliable information to help me form an image of what to expect and to be able to make the right choices for treatment. In this phase, the breast care nurses, whom I could call whenever I wanted and to whom I could go to with all my questions, were very helpful. They had a lot of experience and expertise and were a real source of information for me. That gave me something to hold on to. *(Huijsmans, 2021, 41)*

The patient chooses to enjoy her time while actively seeking information about breast cancer. The pursuit of reliable information not only serves to prepare the patient for ‘what is to come’ but also empowers her to make informed decisions regarding her treatment. The mention of nurses as a valuable resource emphasises the significance of professional support in navigating the uncertainties of waiting.

Having a diagnosis is important because only then a treatment plan can be made. So, with the diagnosis, also comes a decision about the treatment plan which offers patients a sense of control over the waiting:

Then the treatment plan is discussed: until August 2nd I may go on vacation with hormone tablets, on August 3rd there will be a PET scan, August 4th a MRl scan with a biopsy of the right breast, then 6 courses of chemotherapy, surgery and radiation… I'm getting the whole package. It all goes pretty fast. We ask questions and the nurse explains all the follow‐up tests that are going to take place. I feel strong, I'm ‘happy’ with the clarity about what I'm up against. *(Huysmans, 2022, 19)*

Besides treatment plans, in some cases, personal statistical graphs proved to give some sense of control:

It's really nice that the internist is going to make a personal chart with my survival rate. She wants to reassure me with this because I am very afraid that the cancer will come back. The graph indicates that I have more than a 90 percent chance of surviving. That reassures me at least for now. *(Linden, 2022, 52)*

This author finds reassurance in a personalised survival rate graph created by the internist. This representation of the likelihood of survival provides immediate comfort, addressing the patient's fear of cancer recurrence thereby alleviating, albeit maybe only temporarily, the burden of waiting for the cancer.

For some patients more information brings relief. Others find this relief in not knowing or postponing the uptake of certain information. Where in previous paragraphs instances of waiting were mostly imposed on patients, there are cases where patients actively choose to delay receiving certain information or results. For example, in Huysmans’ case (2022), she deliberately postpones learning about her condition until after her planned holiday, demonstrating a conscious choice in timing. This differs from situations where waiting exacerbates fear, instead, in these cases, waiting serves as a strategy for emotional control.

Within the waiting process, there is a significant amount of time of repetition, such as undergoing scans, undergoing surgeries, and spending time in waiting rooms. Over time, patients seem to find ways to harness themselves with ways of navigating time while waiting. Besides seeking information, following or creating routines helps to be ‘in control’.

It is a year after the diagnosis that turned everything upside down. It is unimaginable how focused I was at the beginning, with lists and a countdown calendar, ready for the big battle named chemotherapy. To maintain a sense of control, I could completely put my analytical brain into my homemade countdown calendar. On one page I graded each day, kept track of whether I had managed to exercise, how I had slept and what side effects I was experiencing. On the other page I had a daily schedule to check off: get up, have breakfast, brush teeth, rinse with salt, rub in everything, sleep, repeat. (…) By now I have already lost count, that too is becoming routine. Every three weeks I get up on time, usually to catch up on some sleep on a chair at the infusion in the hospital. It's bizarre how quickly I get used to something like that. *(Zoelman, 2022, 31)*

This quote shows the patient's need for control and structure. The detailed daily routine, even including tasks like tooth brushing and applying lotion, contributes to a feeling of stability amidst the turbulence of cancer treatment. Due to the many repeated experiences, one becomes more advanced in navigating time while waiting, and thereby also the time work they engage in. It is good to realise that while time work is frequently individualistic [22], time work can also mean syncing in with the rhythm of the treatment, in this case, immunotherapy.

Breast cancer patients also demonstrate advanced waiting strategies as they navigate the healthcare system, for example, by actively engaging with healthcare professionals, by proactively seeking certain healthcare procedures like scans and consulting with a specialist or medications. These actions mostly serve as ways to deal with the emotional aspects of waiting like uncertainty and anxiety. In the following example, the patient asks for a scan during her chemotherapy to see how much the tumour shrunk:

Even though I have been told by the oncologist‐internist several times that she feels the tumour is shrinking, I want proof. *(Visser, 2021, 105)*

Additionally, patients utilise peer support networks, exchanging advice and insights with fellow patients who have undergone the same treatments. The following quote illustrates how this waiting work facilitates informed decision‐making, as demonstrated by a patient who sought clarity on treatment options, ultimately leading to the possibility of immediate breast reconstruction:

I also have a lot of contact these days with peers who have already had surgery. They give good advice. One has stuck with me: don't go into the operation with unanswered questions. I notice that I still have quite a few questions. For example, it is not entirely clear to me why I cannot have a direct [breast] reconstruction (…) I have been told that my breast surgeon will contact me today. *(Visser, 2021, 133)*

These examples show the waiting work patients engage in, and it is not only something patients do themselves but also together with others. This waiting work helps to relieve the burden of waiting, although it should be noted that the burden of waiting is always there.

## 4 Discussion

This study aimed to understand the multifaceted nature of waiting by focusing on patients' waiting experiences. The patient stories aptly served the purpose of studying these subjective experiences of time. Books, in contrast to different research methods like interviews, frequently span long timeframes, allowing for a more seamless exploration of waiting experiences. This is interesting as previous research states that waiting is particularly challenging to research [29]. We uncovered three themes that describe the complexity of waiting. The ‘thickening of time’ reveals how moments of waiting hold immense significance, altering the perception of time and intensifying emotions, despite their brevity in clock time. This adds to our understanding of waiting experiences not only as elongated [16] but also as a source of emotional distress. ‘Contaminated time’ highlights the ongoing and continuous nature of waiting throughout the illness process, shedding light on the emotional complexity that arises from emotions like hope and anxiety. This theme illustrates the nuanced and multi‐layered experience of waiting as patients anticipate several aspects beyond care throughout the illness process, in line with the qualitative understanding of waiting [17]. Third, ‘navigating time’ delves into how patients actively engage in managing the practical and emotional challenges of waiting, presenting a specific form of time work [23], namely waiting work. These findings highlight the experiential aspects of waiting, illustrating its complexity and its impact on various facets of care.

Experiences with waiting show to be connected to the organisation of healthcare services. Our research has provided insights into how breast cancer care is structured in the Dutch context, illustrating how patients navigate the rhythm of the hospital system. This rhythm is influenced by the coordination of various healthcare activities, and professionals show to adjust care based on these waiting experiences. For instance, breast cancer nurses offer support that can help ease the stress associated with waiting. However, our analysis also aligns with Day's [5] observation that waiting experiences can expose inefficiencies in healthcare organisation. Our study shows that waiting practices show that rhythms can clash, contributing to patients' burden of waiting. A key example is when a patient's temporal agency is limited, suggesting that healthcare providers and organisations may not always consider the impact of waiting on patient experiences. This lack of alignment can make waiting distressing and disruptive, especially when patients feel they are not receiving care as expected. Analysing patient experiences thus allowed us to explore the concept of 'entrainment' [18], shedding light on how organizations with their rhythms and structures might alleviate the burden of waiting or inadvertently create additional burdens for patients by imposing certain constraints.

It is crucial to recognise that the burden of waiting can stem not only from the processes of the healthcare system but also from the inherent nature of the illness itself. Waiting is, therefore, an inherent part of the burden of disease. Our study demonstrates that waiting is inevitable and cannot be avoided. Nonetheless, there are ways to alleviate this burden of waiting, for instance through the waiting work performed by patients themselves. We have shown the diverse ways in which patients engage in waiting work, recognising that these approaches can vary among patients. 'Waiting work' also shows to evolve during the illness process, for instance, because patients get to know the healthcare system better. This waiting work can be understood as part of broader coping processes, as exemplified by strategies of people with advanced‐stage cancer [13].

This research focused on the experiences of women with breast cancer. The life‐threatening aspect of this disease contributes to the waiting experiences, adding another layer of complexity to the patient's journey. This study aligns with earlier findings on the emotional impacts of certain procedures. For instance, cancer patients often experience high levels of anxiety during scans, a phenomenon commonly referred to as 'scanxiety' [30]. Similarly, uncertainty about medical results can lead to varied emotional responses among patients. While some may experience anxiety over a potential nodule, others may find themselves in a state of equanimity [31]. Although some waiting experiences are uniquely associated with breast cancer, other elements, such as emotional responses and waiting work, are likely shared by patients facing other life‐threatening diseases. This implies that our findings could be relevant across a range of other diseases.

### 4.1 Limitations

It is important to acknowledge that our analysis may not encompass the full spectrum of waiting experiences, including waiting work undertaken by patients. This limitation arises from our selected data set, in which there is some selection bias. It is important to realise that patients who choose to write about their experiences are not representative of the entire patient population. Waiting experiences can vary, as the temporal agency is also tied to one's social capital [22], and other factors such as socioeconomic status, gender, and discrimination may also shape these experiences. Additionally, factors like religion may further nuance how waiting work is performed by patients. Although these elements were not explored in our analysis, they offer important directions for future research to provide a more comprehensive understanding of the waiting experiences across different patient groups.

### 4.2 Future Research

Further research should focus on other waiting experiences in other conditions and circumstances. For instance, waiting experiences could differ between diseases. Diseases for which it is hard to get a diagnosis and treatment, for instance in the case of ME/CFS [32] and long COVID [33] are especially interesting to study with regard to waiting. This would add more to our understanding of the qualitative aspect of waiting for patients.

### 4.3 Lessons for Practice

Besides having a logistical and a policy component, waiting is very much an intense experience for patients throughout their disease. While actions for reducing waiting lists and waiting times are important, our research also revealed that the burden of waiting, as Salisbury et al. [2] also argue, depends not only on its quantity but also on its quality. Therefore, gaining a deeper understanding of patients' waiting experiences—whether they are on waiting lists or already undergoing treatment—is crucial. The waiting functions as a signal for the quality of care, which shows that there are valuable lessons to be drawn from these experiences for improving the quality of care and support by working towards more patient‐centred care. Our analysis shows the clashes of rhythms between patients and the organizations of care can result in negative experiences for patients. Acknowledging a patient's distress while waiting is important during waiting moments, as patients deal with intensified emotions and distress, which could be relieved by being more sensitive towards patients' temporal experiences.

## Author Contributions

**Nada Akrouh:** conceptualisation, investigation, writing–original draft, methodology, writing–review and editing, formal analysis, data curation. **Rik Wehrens:** writing–review and editing, supervision, methodology. **Erna Scholtes:** investigation, formal analysis, writing–review and editing, methodology. **Hester Bovenkamp:** writing–review and editing, supervision, methodology.

## Ethics Statement

Ethical review and approval were not applicable to this study as it involved the analysis of publicly available data. To ensure transparency, we contacted the original authors or publishers of the materials used in our analysis to inform them of our research and the inclusion of their works.

## Conflicts of Interest

The authors declare no conflicts of interest.

## Supporting information

Supporting information.

## Data Availability

Data supporting the findings of this study are referenced in the supplementary material of this article. However, access to the data is restricted due to copyright laws, as the data consists of books protected by authorship rights.
