# Supplementary material for: Beyond the Queue: Exploring Waiting Practices in the Stories of Patients With Breast Cancer
Source: Health Expect. 2024 Nov 17;27(6):e70086. doi: 10.1111/hex.70086 (PMC11570769; doi:10.1111/hex.70086)
Supplement: Supplementary file 1 — Supporting information. [file HEX-27-e70086-s001.docx]

| Author | Title | Publisher | Year of publication | Number of pages | Link to description book |
| --- | --- | --- | --- | --- | --- |
| Caroline Reeders | U mag even plaatsnemen | Nijgh & van Ditmar | 2021 | 176 | <https://www.patientervaringsverhalen.nl/collectie/u-mag-even-plaatsnemen/> |
| Dorothé Huijsmans | Gelukkig kreeg ik kanker | Brave New Books | 2021 | 136 | <https://www.patientervaringsverhalen.nl/collectie/gelukkig-kreeg-ik-kanker/> |
| Jannie Oskam | Tussenland | Uitgeverij de Graaff | 2021 | 160 | <https://www.patientervaringsverhalen.nl/collectie/tussenland/> |
| Gerdie Bours-Willems | Dwars door de berg | Uitgeverij Elikser | 2021 | 294 | <https://www.patientervaringsverhalen.nl/collectie/dwars-door-de-berg/> |
| Eva Visser | Huisje, boompje... Borstkanker | Growing stories | 2021 | 172 | <https://www.patientervaringsverhalen.nl/collectie/huisje-boompje-borstkanker/> |
| Kitty Trepels van Mil | De vrouw in 20 wachtkamers | De Vrije Uitgevers | 2022 | 74 | <https://www.patientervaringsverhalen.nl/collectie/de-vrouw-in-20-wachtkamers/> |
| Helma Huysmans- Van Atteveld, Edith Idoe-Stap | Kanker en Werk | Uitgeverij Zilt | 2022 | 176 | <https://www.patientervaringsverhalen.nl/collectie/kanker-en-werk/> |
| Dianora Porru | Door de tunnel | Boekscout | 2022 | 162 | <https://www.patientervaringsverhalen.nl/collectie/door-de-tunnel/> |
| Marloes Scheffers | Wie is de held? | Boekscout | 2022 | 130 | <https://www.patientervaringsverhalen.nl/collectie/wie-is-de-held/> |
| Eline Zoelman | Zuurtjes en zalfjes | Boekscout | 2022 | 102 | <https://www.patientervaringsverhalen.nl/collectie/zuurtjes-en-zalfjes-palliatieve-kankerzorg-op-je-34e/> |
| Monique van der Linden | De amputatie heeft stilte plaatsgevonden | Boekscout | 2022 | 126 | <https://www.patientervaringsverhalen.nl/collectie/de-amputatie-heeft-in-stilte-plaatsgevonden/> |
| Marjolein van Oosterbos | Het jaar dat ik kind en kanker kreeg | Boekscout | 2023 | 166 | <https://www.patientervaringsverhalen.nl/collectie/het-jaar-waarin-ik-een-kind-en-kanker-kreeg/> |

*Table S1: included books*
